# Head‐to‐head comparison of four different plasma p‐tau217 assays to detect brain amyloid pathology and cognitive decline in diverse population‐based cohorts

**DOI:** 10.1002/alz.092871

**Published:** 2025-01-09

**Authors:** Rebecca A Deek, Xuemei Zeng, Fernando Gonzalez‐Ortiz, Wasiu Gbolahan Balogun, Pamela C.L. Ferreira, Bruna Bellaver, Yijun Chen, Anuradha Sehrawat, Tara K Lafferty, Guilherme Povala, William E Klunk, M. Ilyas Kamboh, Victor L Villemagne, Tharick A. Pascoal, Mary Ganguli, Henrik Zetterberg, Kaj Blennow, Oscar L. Lopez, Ann D Cohen, Beth E. Snitz, Thomas K Karikari

**Affiliations:** ^1^ University of Pittsburgh, Pittsburgh, PA USA; ^2^ Institute of Neuroscience and Physiology, University of Gothenburg, Mölndal Sweden; ^3^ Department of Psychiatry, University of Pittsburgh School of Medicine, Pittsburgh, PA USA; ^4^ University of Pittsburgh Alzheimer's Disease Research Center (ADRC), Pittsburgh, PA USA; ^5^ School of Public Health, University of Pittsburgh, Pittsburgh, PA USA; ^6^ Division of Geriatrics & Neuropsychiatry, Department of Psychiatry, University of Pittsburgh, Pittsburgh, PA USA; ^7^ Department of Psychiatry and Neurochemistry, Institute of Neuroscience and Physiology, The Sahlgrenska Academy, University of Gothenburg, Mölndal, Gothenburg Sweden; ^8^ Institute of Neuroscience and Physiology, The Sahlgrenska Academy at the University of Gothenburg, Mölndal Sweden; ^9^ Department of Psychiatry, School of Medicine, University of Pittsburgh, Pittsburgh, PA USA

## Abstract

**Background:**

Plasma p‐tau217 is a highly promising biomarker for detecting Alzheimer’s disease (AD) pathology. However, it is unclear how p‐tau217 assays from different sources perform compared to one another. Moreover, studies in diverse cohorts and population‐based settings are limited. We performed a direct comparison of four different p‐tau217 assays to detect brain amyloid‐beta (Aβ) deposition or cognitive decline.

**Method:**

We included n=427 participants from three separate cohorts from southwestern Pennsylvania, USA. Cohort‐1 was the Monongahela‐Youghiogheny Health Aging Team (MYHAT, n=87, median age 83 [IQR: 78‐87.5], 74% female), an ongoing population‐based cohort drawn from a medically and economically underserved Rust Belt region. Cohort‐2 was the MYHAT‐NI sub‐study (n=111, median age 76 [IQR: 72‐80], 54% female), which performed two‐year longitudinal follow‐up neuroimaging assessments of Aβ, tau, and neurodegeneration by structural MRI. Cohort‐3 was the Human Connectome Project (HCP, n=229, median age 62 [IQR: 56‐70], 65% female) which targeted a 50:50 split of self‐identified Black and non‐Hispanic White individuals. Plasma p‐tau217 was measured using assays from four different sources: University of Pittsburgh (UPitt), ALZpath, University of Gothenburg (UGOT), and NULISA from Alamar Biosciences. Aβ positivity was defined as a mean SUVR > 1.346. Clinical Dementia Rating (CDR) and Mini Mental State Examination were used to assess cognitive performance.

**Result:**

In the MYHAT‐NI cohort, three p‐tau217 assays—UPitt, ALZpath, and NULISA—were employed. The assays demonstrated equivalent AUCs for determining Aβ positivity, ranging from 0.84 to 0.90. UPitt and ALZpath assays also showed similar AUCs in the HCP cohorts, with values of 0.87 and 0.90, respectively. In MYHAT, the UGOT and ALZpath assays separated CDR=0 and CDR=0.5 participants with equivalent AUCs (0.79 vs. 0.81). Significant correlations were observed in all inter‐assay comparisons, with Pearson correlation between assays ranging between 0.40 and 0.86.

**Conclusion:**

We observed largely equivalent performances of plasma p‐tau217 assays in predicting amyloid positivity and cognitive impairment across three diverse cohorts of community‐dwelling older adults.

